# “Left Speechless in Front of My Children”: A Thematic Analysis of Parents’ Experiences During War & Displacement

**DOI:** 10.1007/s40653-026-00974-4

**Published:** 2026-09-04

**Authors:** Tamar Hadar

**Affiliations:** School of Creative Arts Therapies, University of Haifa, Haifa, Israel

**Keywords:** War, Displacement, Parenting, Trauma, Hosting-community

## Abstract

The October 7, 2023, terrorist attack in Israel and ensuing mass displacement created acute, unprecedented psychological crises for families. While the impact of war and forced migration on children is well documented, far less is known about how parents, especially mothers, sustain caregiving under such extreme, ongoing threat. This study explores the lived experiences of displaced Israeli mothers, focusing on the emotional, relational, and functional dimensions of parenting during and after trauma. In-depth, semi-structured interviews were conducted with four Israeli mothers displaced with their children following the attack. Interviews were transcribed verbatim and analyzed thematically using an inductive approach, with particular attention to emotional narratives, caregiving practices, and perceptions of both destroyed homes and hosting environments. Three themes emerged: (A) Alone in the Shelter: The Weight of Solo Parenting During Attack, War, and Displacement; (B) From Regulated Parenting to Fragmented Parenting; and (C) The Double-Edged Sword of the Hosting Community. Together, they depict intense emotional strain, destabilized parental identities, children’s accelerated maturity or increased dependency, and ambivalent experiences of host communities as simultaneously protective and destabilizing. Parenting in this context entails both relentless protection and gradual emotional erosion. Mothers oscillated between composure and collapse, often with limited systemic support. The findings underscore the need for trauma-informed, relationally grounded interventions that recognize caregivers as survivors in their own right and prioritize their emotion regulation, reflective functioning, and restoration of parental coherence as central to family recovery.

The October 7, 2023, terrorist attack in Israel and the subsequent mass displacement of civilians created an unprecedented rupture in family life, caregiving routines, and psychological safety. While research on the impact of war and forced migration has increasingly highlighted children’s vulnerability (Bürgin et al., 2022; Hazer & Gredebäck, 2023), far less attention has been given to the lived experiences of parents, particularly mothers, tasked with caregiving under extreme stress, uncertainty, and separation. Parental distress has been identified as a key predictor of family functioning during crises (Alfasi et al., 2025), and solo parenting has emerged as a particularly acute risk factor. Mothers often serve as both emotional regulators^1^ and protective buffers for their children, yet their own narratives of overwhelm, role fragmentation, and relational breakdown remain underexplored (Feldman & Vengrober, 2011; Gillespie et al., 2022). Similar gaps have been noted in Middle Eastern and Palestinian scholarship, where parents in Gaza, the West Bank, and neighbouring conflict zones shoulder immense emotional and practical burdens while attempting to protect children under siege, missile strikes, and prolonged displacement (Akesson & Sousa, 2020; Hamamra et al., 2025; Thabet et al., 2008). This study explores how Israeli mothers navigated parenting in the wake of terror and dislocation, illuminating the emotional, relational, and functional strains placed on caregivers during and after the crisis. Through in-depth qualitative interviews and thematic analysis, the study examines how trauma, loss of home, and fractured social supports reconfigured parental identity, emotional regulation, and parent-child dynamics.

## Parenting in Times of War & Trauma

War significantly increases parental stress, leading to behaviors such as overprotection and reduced emotional responsiveness towards children (Conway et al., 2013). This stress is further aggravated by displacement, financial strain, and disrupted family roles, making it difficult for parents to provide stability (Bayer & Shtudiner, 2023; Wang & Pullman, 2019). War exposure can also lead to mental health issues like PTSD and depression in parents, which negatively affect their ability to regulate both their own and their children’s emotions 

(Eltanamly et al., 2021). Consequently, this can result in harsher parenting, less warmth, and increased hostility in parent-child interactions (Suardi et al., 2017). Beyond these effects on parenting behaviour, recent research emphasizes that parents’ perceptions of war are themselves clinically meaningful. In a qualitative study of Israeli parents during the first month of the war following the October 7 attack, parents described war through experiences of shock, uncertainty, alertness, abandonment, helplessness, fear for themselves and others, and concern about the unknown future; they also evaluated their own parental functioning under conditions of acute complexity and uncertainty (Mijalevich-Soker & Taubman-Ben-Ari, 2025). The importance of parenting practices in either alleviating or intensifying the effects of mass trauma on children’s adjustment has been emphasized in various studies (Joffe et al., 2025; Gewirtz et al., 2008 Hoffman & Shrira, 2019; Möllerherm et al., 2025; Murphy et al., 2017; Pat-Horenczyk et al., 2012). Drawing on a broad range of studies, Murphy et al. (2017) examined the intricate dynamics of family relationships in the context of war and explored how war trauma impacts not only parents’ mental health but also their parenting practices, which, in turn, influence the psychological and emotional well-being of children. The authors noted that parents who have experienced traumatic war events may exhibit significant changes in their behaviour and parenting styles. Such experiences can result in increased authoritarianism, hesitation, and withdrawal in parent-child interactions. Conversely, some mothers, after encountering traumatic events, may become less strict and exercise less control, thereby affecting the dynamics of mother-child interactions.

Several researchers (e.g., Joffe et al., 2025; Khamis, 2016; Mladenović et al., 2021) demonstrated that parental psychological distress acts as a mediator in the relationship between war trauma and children’s emotional and behavioural disorders; showing how psychological distress of parents can predict the likelihood of disorders in children. In research examining the long-term impact of a traumatic event like the 9/11 attacks (Chemtob et al., 2010), the authors not only highlighted its effect on parents but also underscored the severe psychological consequences for their children. The study emphasized the need to address the intergenerational transmission of trauma effects, particularly noting that children are at greater risk of psychological distress when exposed to a combination of maternal depression and PTSD. At the same time, parenting should be understood not only as a site of impact, but also as a potential mediator of child outcomes. War-related parental distress may compromise warmth, emotional availability, routines, and discipline, thereby transmitting or intensifying children’s distress. Conversely, sensitive, predictable, and emotionally regulated caregiving may buffer children’s exposure to danger and support resilience (Gewirtz et al., 2008; Murphy et al., 2017; Pat-Horenczyk et al., 2012).

The developmental impact of trauma in early childhood cannot be understood solely in relation to the precipitating event. Whether the threat originates in war, forced displacement, interpersonal violence, neglect, or frightening caregiving, early trauma may compromise the attachment^2^ and relational systems through which the child ordinarily develops a sense of safety, trust, and emotional regulation. Trauma in early childhood simultaneously assaults the attachment system and the developing brain (Schore, 2009). Repeated experiences of fear, inconsistency, or frightening caregiving disrupt the child’s expectation of the caregiver as a safe, emotionally available figure, increasing the likelihood of insecure or disorganized attachment and weakening the capacity to use adults as a secure base for exploration and regulation. In parallel, chronic stress reshapes neural circuits involved in threat detection and emotion regulation (e.g., amygdala, hippocampus, prefrontal cortex), leading to patterns of hyperarousal or dissociation that interfere with attention, learning, and integration of experience. Together, these relational and neurobiological disruptions compromise children’s emerging sense of self, their ability to regulate affect, and their developmental trajectories across emotional, social, and cognitive domains (Díaz et al., 2024; Lanius et al., 2018; Perl et al., 2023; Ressler et al., 2022; Schore, 2009).

Trauma, especially chronic interpersonal violence and frightening or inconsistent caregiving, attacks attachment by shattering the young child’s expectation that the caregiver is protective, emotionally available, and reliably responsive, thereby destabilizing early attachment bonds (Lieberman & Van Horn, 2005; Lieberman & Horn, 2008). Over time, such experiences are internalized as fear and confusion within the attachment system, increasing the likelihood of insecure or disorganized attachment patterns and limiting the child’s ability to use caregivers as a secure base for exploration and affect regulation (Lieberman & Van Horn, 2005; Lieberman & Horn, 2008).

Regional work among Israeli and Palestinian families further highlights how political violence and chronic insecurity penetrate family systems. Longitudinal studies with Israeli and Palestinian youth show that exposure to ethnic and political violence increases parental depression and interparental aggression, which in turn heighten harsh physical punishment and predict later youth aggression (Dubow et al., 2025). In Gaza, parents and children frequently report high levels of trauma exposure, PTSD symptoms, and anxiety (Thabet et al., 2008), with qualitative work depicting mothers and fathers as “guardians of the night,” monitoring danger and soothing distressed children while coping with their own sleep disruption and fear (Hamamra et al., 2025). Among recently displaced Syrian and Palestinian refugee parents, studies similarly describe intense psychological suffering, grief, and loss of parental efficacy, alongside a strong moral commitment to protect, comfort, and guide children through war and displacement (Akesson & Sousa, 2020).

## Parenting & Displacement

Parenting under conditions of forced displacement presents a complex set of emotional, relational, and environmental challenges. A growing body of research highlights the potential of caregiver-focused interventions to support families experiencing displacement; however, findings suggest that these programs often achieve only partial success: While some interventions have shown improvements in maternal well-being and reduced caregiver distress (Dybdahl, 2001; Morris et al., 2012; Ponguta et al., 2020), their impact on broader relational dynamics, such as parental responsiveness, discipline practices, and children’s emotional and behavioral adjustment, remains inconsistent (Stark et al., 2018).

This variability points to a need for parenting programs that go beyond behavioral strategies to address deeper emotional processes, including parental emotion regulation and stress management (Gillespie et al., 2022). Importantly, effective support must also consider families’ basic needs, such as safety, food, and access to medical care, especially in the context of acute or prolonged crisis, a need often overlooked in high-income settings.

The current research focuses on the influence of war and displacement on parenting, aiming to explore the layered emotional, psychological, and relational effects of trauma and displacement on parents, particularly mothers. Through a qualitative lens, the current study sought to uncover how war-related upheaval reshaped caregiving capacities, emotional availability, and parent-child relationships in the aftermath of the October 7 attack. Rather than assessing the outcomes of specific interventions, the goal was to give voice to parents’ lived experiences, highlighting the intersections between personal distress, disrupted routines, and the ongoing effort to maintain a sense of connection and safety for their children amidst uncertainty.

### The Current Research

On October 7, 2023, Hamas launched a brutal terrorist attack on Israeli communities near the Gaza border, resulting in over 1,200 deaths and the abduction of more than 250 individuals (Palgi et al., 2024). Since the attack, studies have reported a sharp rise in psychological distress among Israelis, including increased rates of PTSD, anxiety, depression, and suicidal ideation (Levi-Belz et al., 2024).

There is an abundant literature concerning the devastating effect of war on children’s social, emotional and cognitive states (Beehler et al., 2012; Betancourt et al., 2012; Hazer & Gredebäck, 2023). However, the impact of war-related experiences on parenting and consequently the role of parenting practices in mediating the overall influences of war on children’s well-being has been significantly less discussed in the literature. This gap is particularly striking when contrasted with the rapidly growing body of regional work documenting how Israeli, Palestinian, and Syrian parents attempt to hold families together under bombardment and displacement (Akesson & Sousa, 2020; Dubow et al., 2025; El-Khani et al., 2017; Hamamra et al., 2025; Thabet et al., 2008).

The current study aims at filling this gap by exploring the views of parents who experienced extreme war events and displacement for six months. Examining parents’ firsthand experiences may clarify these families’ needs and inform the development of parent-focused interventions for contexts of war and displacement. The research questions included:

1. How do parents who experienced an extreme terror attack followed by displacement perceive their parenting and their relationship with their children in this period of time?
2. What points of strength/challenge were described by parents in light of their extreme experience?

## Methods

### Reflexivity and Epistemological Stance

This study is situated within an interpretivist–constructivist epistemological framework (Crotty, 1998). From this perspective, participants’ accounts are understood as situated narratives through which lived, embodied, relational, and affective meanings are articulated. The study is concerned with understanding how participants’ experiences in the context of trauma and displacement. Accordingly, the themes are viewed as interpretive constructions generated through participants’ narratives, the interview context, and the researchers’ reflexive engagement with the data (Crotty, 1998).

The author’s reading of the material is shaped by her positionality as an Israeli, white, Western-trained music therapist, and by her own proximity to the events of October 7, 2023, and their aftermath. The author did not approach the interviews as a neutral observer standing outside the traumatic reality described by participants, but as a researcher and clinician whose professional, national, emotional, and embodied knowledge inevitably informed the analytic process. In the months following the attack, the author worked as a music therapist with displaced families in Israel, an experience that deepened her sensitivity to the ways trauma can be held not only in words, but also in silence, bodily states, ruptured relational patterns, and altered musical presence.

This proximity was both a resource and a limitation. It enabled an attuned understanding of the socio-political, familial, and therapeutic contexts in which participants’ experiences unfolded, while also requiring ongoing reflexive attention to the assumptions, identifications, and emotional resonances brought to the analysis. From this epistemological stance, the findings are not presented as objective truths discovered in the data, but as situated interpretations generated through participants’ narratives, the research encounter, and the author’s own reflexive engagement with the material. This does not diminish the reality of the traumatic events or their consequences; rather, it acknowledges that the meanings of trauma and displacement are understood through relational, cultural, historical, and embodied lenses.

### Context

This study involved four families who were displaced from their home kibbutz near the Gaza border following the October 7, 2023, terror attack by Hamas. The families were temporarily relocated to a northern village in Israel as part of a national emergency rehabilitation program.

A clinic was created specifically for members of the displaced kibbutz and provided therapeutic support to families during the period of displacement. Most families in the community attended therapy, although the duration of treatment varied: some received only a small number of sessions, whereas others participated in more extended therapeutic interventions. The therapy sessions offered a supportive and containing context in which families could begin to approach and process their recent experiences. Although the present study did not aim to evaluate the intervention itself, the mothers’ recent engagement in therapy was methodologically relevant: it was understood as a form of emotional scaffolding that could support their capacity to revisit, articulate, and reflect upon highly distressing parenting experiences without becoming overwhelmed.

### Recruitment

All families were recruited via the temporary clinic established to support the displaced families who arrived from the border area. It was staffed by ten volunteer therapists and provided services to approximately 40 families, with some families receiving multiple forms of support, including individual therapy for children, parental support, and family-based interventions. A few weeks after therapy has ended, families were approached by their therapist and asked if they are willing to take part in the study. Therapists were handed an information form explaining that the study, led by Dr Tamar Hadar from the University of Haifa, aims to explore how the October 7, 2023, Hamas terrorist attack and its aftermath impacted the parenting experiences of families who were displaced and received therapeutic support for their young children (ages 3–9). Eligible participants must have ended therapy at least two months before participating.

To minimize bias in referrals, therapists were instructed to consider all families who met these eligibility criteria and appeared emotionally stable enough to participate, rather than selectively approaching particular parents. Therapists were asked to assess emotional readiness and to avoid referring individuals who exhibited significant post-traumatic symptoms during therapy, were still in treatment, or had prior therapeutic contact with the researcher. Because therapy had already concluded, and therapists explicitly emphasized that participation was entirely voluntary and unrelated to past or future services, the risk of coercion or perceived obligation was reduced. Parents could choose either to contact the researcher directly or to authorize their therapist to forward their contact details, thereby further separating the clinical and research roles.

### Participants

Six therapists were approached. Identifying eligible families was challenging, as many of the families were experiencing severe post-traumatic stress symptoms and were therefore not considered suitable for participation in an interview. Three therapists reported that they had no eligible families, either because the families were still receiving therapy or because the children were experiencing symptoms of a severity that precluded participation. The remaining three therapists, all working at the temporary clinic, were contacted by the researcher. The first therapist, a music therapist, approached two families who appeared eligible for the study; one agreed to participate. The second therapist, an art therapist, approached one family considered suitable for inclusion, and the family accepted the invitation. The third therapist, a dance-movement therapist, approached two eligible families, both of whom agreed to participate. Ultimately, four families met the inclusion criteria, were judged emotionally ready, and consented to participate in the study (see Table 1).

**Table 1:** Participants’ Demographics

Pseudonym	Age	Marital Status	Profession	# of Children
Miri	39	Married	Finance	4
Yafa	41	Married	Marketing	3
Rony	33	Married	Housewife	3
Yuli	36	Married	ER Nurse	3

### Data Gathering and Analysis

Semi-structured interviews were conducted with each of the four participating Israeli mothers at the conclusion of a short-term music therapy intervention following their displacement during the October 7, 2023, attack. The study involves a one-time, semi-structured interview, during which parents were invited to reflect on their parenting during the war and on the parental guidance they received.

The interviews were conducted in Hebrew and ranged from 60 to 90 min, all conducted via Zoom. This format allowed participants the freedom to speak in their native language about their lived experiences of parenting under conditions of trauma and forced displacement. An open-ended, non-interventionist interview style was used to facilitate a rich, in-depth understanding of participants’ experiences and perceptions of the therapy’s impact, on themselves, on their children, and on their parenting. The topics covered in the interviews included, but were not limited to, the family’s experience of October 7; the transition to their new temporary dwelling; life in displacement; reflections on parenting; perceptions of their children’s experiences; differences among children and their roles within the family; and the impact of displacement on family structure and well-being. The author deployed Braun and Clarke’s (2022) six-phase approach: familiarization, coding, theme development, theme review, theme naming, and report writing. This inductive method emphasizes close engagement with the data to identify recurring patterns or themes without imposing a prior theoretical framework.

The process unfolded in the following steps:

1. Transcription: All interviews were transcribed verbatim in Hebrew by the first author. Identifying information was removed during transcription to preserve confidentiality.
2. Familiarization: Transcripts were read multiple times by each researcher to gain a deep understanding of the content, accompanied by analytic memo writing.
3. Coding: Segments of text were coded and color-coded based on content similarities, with particular attention to emotional, relational, and contextual nuances.
4. Theme Identification: Codes were clustered to form preliminary themes that captured key ideas expressed across participants.
5. Theme Review: The first author discussed her analysis with an external consultant who has expertise in thematic analysis. Together they further highlighted patterns across transcripts, refining categories and resolving discrepancies.
6. Naming Themes: Final themes were defined and named to reflect the depth and scope of the narratives.
7. Final Integration: The first author integrated insights from all three researchers into a final thematic structure, which is presented in the Findings section.

### Ethical Considerations

This study received ethical approval from the Institutional Review Board of the Institutional Review Board the Welfare and Health Sciences at the University of Haifa (#345/24), ensuring adherence to established ethical standards for research with human participants. Informed consent was obtained from all participating parents. Participants were assured of their right to withdraw at any stage without consequence. Given the sensitive nature of war-related trauma and displacement, particular care was taken to protect participant wellbeing, including offering optional debriefing conversations and ensuring that all interviews and sessions were conducted in safe, supportive environments. Identifying information was anonymized in transcripts and publications to safeguard confidentiality.

### Findings

Analysis of the interview data from four mothers who experienced displacement following an extreme terror attack revealed three overarching themes and several related subthemes (see Table 2). Together, these themes illuminate the mothers’ lived experiences of parenting during attack, war, and displacement, as well as the ways in which these circumstances affected their parental roles, emotional availability, and sense of stability. The first theme, *“Alone in the Shelter”: The Weight of Solo Parenting During Attack*,* War*,* and Displacement*, captures the burden of caring for children amid acute danger and prolonged uncertainty. It includes two subthemes: “My Child Grew Up Overnight”: Shifting Parent-Child Roles, which reflects the ways in which children appeared to mature rapidly or assume altered roles following the attack; and Heightened Dependency and Lack of Separation, which describes children’s intensified need for proximity and reassurance.

**Table 2:** Themes & Subthemes

Themes	Subthemes
*“Alone in the Shelter”: The Weight of Solo Parenting During the Attack*,* War and Displacement*	*“My Child Grew Up Overnight”: Shifting Parent-Child Roles* *Heightened Dependency and Lack of Separation*
*From Regulated Parenting to Fragmented Parenting*	*Emotional Spillover and Parental Overwhelm: “You Feel Like a Failure as a M* *other!”* *Fragmented Presence: Caregiving While Absent* *Parental Helplessness: “I Was Left Speechless"*
*The Double-Edged Sword of the Hosting Community: A Stabilizing Factor in a Disastrous Context*	*Challenges of Displacement: Overcrowding*,* Stress*,* and Uncertainty* *A Refuge That Could Not Hold: Ambivalent Safety in the Hosting Community*

The second theme, *From Regulated Parenting to Fragmented Parenting*, addresses the disruption of mothers’ capacity to remain emotionally available and regulated under conditions of ongoing stress. It comprises three subthemes: Emotional Spillover and Parental Overwhelm: “You Feel Like a Failure as a Mother!”; Fragmented Presence: Caregiving While Absent; and Parental Helplessness: “I Was Left Speechless.”

The third theme, *The Double-Edged Sword of the Hosting Community: A Stabilizing Factor in a Disastrous Context*, explores the receiving community as both a vital source of support and a setting marked by instability and strain. It includes two subthemes: Challenges of Displacement: Overcrowding, Stress, and Uncertainty; and A Refuge That Could Not Hold: Ambivalent Safety in the Hosting Community. Each theme and its related subthemes are elaborated below.

### Alone in the Shelter: The Weight of Solo Parenting During Attack, War, and Displacement

For three participants, the trauma of October 7 and the prolonged period of displacement were compounded by periods of solo parenting.^3^ These mothers navigated the crisis while carrying primary responsibility for their children without the physical presence of a partner, an experience that intensified their emotional and logistical burdens. Their narratives reveal not only acute vulnerability but also the activation of powerful caregiving instincts under extreme pressure. These stories underscore how the absence of a partner refracted through moments of existential fear, exhaustion, and decision-making isolation.

Rony, a mother of three children under the age of 10 at the time of the October 7 attack, vividly described the terror of being alone with her children under fire, invoking a prayer she did not customarily say - a moment of involuntary spirituality in a situation beyond comprehension:

*You hear gunfire*,* sheer terror*,* real terror… I was alone in the shelter with three children*,* and my son was in complete hysteria… The girls were crying*,* and I was scared. I had never felt that way before. I found myself saying ‘Shema Yisrael’… something that just came out of me… If my husband had been with us*,* I am sure we would have experienced it entirely differently.* (Rony)

Her reflection contrasts a self-perception of calm competence with an admission of fear and helplessness, exposing the psychological rupture the event caused. The absence of her partner is imagined not only as a logistical gap but as a missing emotional anchor - a counterfactual presence that might have buffered the intensity of her fear.

Yafa, a mother of three children under the age of 11 at the time of the October 7 attack, who remained without her partner for most of the displacement period, portrayed solo parenting as both a structural and emotional weight. Her transition from a full-time professional to sole caregiver reconfigured her identity, producing daily encounters with despair:

*Suddenly*,* I had to manage everything alone. I had to make all the decisions. I remember opening my eyes in the morning and feeling hopeless. Not to mention that I barely slept at night. But every morning*,* it was like*,* damn*,* I have to get through another day… The absence*,* it’s constant… You talk on the phone with your partner*,* but it’s not the same… You are alone*,* with children who miss their father*,* who are struggling… That’s the hardest part*,* the separation*,* the split from your partner.* (Yafa)

Her use of the phrase “the absence, it’s constant” encapsulates the participant’s pervasive sense of her partner’s absence. This contrasts starkly with her former dual-career dynamic and underscores how crisis conditions strip away support structures that once felt stable.

### “My Child Grew Up Overnight”: Shifting Parent - Child Roles

Crisis proximity and the shared intensity of war appeared to catalyze role reversals or accelerated emotional development in children. Interviewees described moments where children took on heightened responsibility or emotional insight, stepping into roles that blurred the line between child and adult. Miri, a mother of four children under 6 years old at the time of the October 7 attack, recounted how candid conversations about death brought her closer to her son but also signaled a loss of childhood innocence: *“Suddenly*,* I realized that something had changed. He had grown up. He became more mature*,* more aware.”* Yuli, a mother of three children under 14 years old at the time of the October 7 attack, shares about her son, who became strikingly self-sufficient during evacuation: *“He woke up in the morning*,* prepared things for himself… He was by himself*,* ate by himself*,* he just took care of himself.* When the attack began, Yuli was immediately called to the hospital where she worked as an ER nurse, while her husband was recruited to the emergency team fighting the terrorists. While these accounts speak to children’s adaptability and emerging resilience, they also carry an undertone of loss of protection, of dependency, and of the developmental space normally reserved for childhood.

In a more complex narrative, Yafa described her son’s emotional eruption, which simultaneously expressed personal grief, moral discernment, and community ambivalence:

*He cried… saying that this was awful*,* that families shouldn’t live this way… ‘I want to go to that host kibbutz and tell them they are awful. I hate them. I hate being here. But I won’t tell them that*,* because… I know I need to stay here*. *And all I could do was hug him and tell him I understood that it was really hard*,* but that this was our reality. There was nothing else I could do for him. It wasn’t enough. He cried himself to sleep from sheer exhaustion. It was devastating.* (Yafa)

This outburst reflects a layered awareness: he both recognizes the necessity of their current living arrangement and resents its emotional cost. His ability to withhold his anger in order to protect a fragile arrangement underscores a premature emotional labor not typically expected of children. Yafa’s response, limited to physical comfort and affirmation, reveals a parallel helplessness: a parent’s pain in acknowledging a child’s suffering without being able to fully alleviate it.

#### Heightened Dependency and Lack of Separation

Alongside narratives of premature maturation, some parents described a regression into dependence. For these families, proximity became claustrophobic, as children clung to parents for safety and regulation long after the immediate danger had passed. Yafa shared:

*When I went to sleep with them in the shelter on the seventh*,* I didn’t realize that I would still be sleeping with them seven or eight months later. You lose your privacy*,* both physically and emotionally.* (Yafa)

This extended physical closeness, while understandable in trauma’s wake, also eroded boundaries and complicated the return to normal routines. The coexistence of premature maturity and prolonged dependency across interviews illustrates a key tension: trauma can fracture developmental trajectories in divergent directions, pushing some children toward hyper-independence and others toward regressive attachment - sometimes both within the same family system.

#### From Regulated Parenting to Fragmented Parenting

Participants described a shift from a familiar sense of parental coherence and emotional regulation toward experiences of overwhelm, fragmentation, and disrupted parental continuity. All participants described reaching emotional breaking points during or after the acute phase of the attack and evacuation. These breakdowns occurred not at the moment of direct threat, but later, once relative safety had been attained.

Miri shared how she shielded her children from the event, a skill she had practiced even before October 7th:

*No. No*,* I think the children were very well protected. They were hardly exposed at all. The nature of the event matters a lot. There’s a difference between experiencing a rocket alert inside your house versus outside. Are you exposed to other people*,* to panic*,* to anxiety? Or are you in a calm environment? We were both very*,* very calm.* (Miri)

In contrast, other parents described their emotional breakdowns once they reached safety, allowing themselves to feel all the emotions they had suppressed during the acute crisis. Yafa recounted:

*The moment I arrived at the evacuation site; I started crying like crazy. I had been holding it together*,* staying very calm*,* until then… And then suddenly*,* over something trivial*,* some random thought*,* I just broke down. Completely out of proportion… it was just this massive release… I had gone from being in control to suddenly losing it all at once.* (Yafa)

This quote reflects the complex layering of roles and expectations placed on parents in extreme circumstances. Yafa’s emotional collapse, triggered by “something trivial,” illustrates how hyper-regulated states often give way to uncontrollable affect once the immediate need for vigilance passes. Her description also conveys a dissociation between the magnitude of her feelings and the specific trigger, suggesting that the breakdown was not about one moment but an accumulation of suppressed fear, responsibility, and exhaustion.

Rony similarly reflected on the painful dissonance between how she wished to appear to her children and how she ultimately reacted: *The children really saw me for the first time very*,* very scared. I never… in my life*,* I have never reacted in such a way*. This moment seems to mark a rupture in her parental identity, one in which the protective facade cracked, revealing her own fear. The emotional cost of trying to be a source of strength, only to “fail” in that attempt, exposes the unattainable expectations parents may carry during disaster. Here, vulnerability was not chosen but emerged despite her efforts. Yuli’s account adds yet another dimension, shifting the focus from visible collapse to emotional numbness and fragmentation:

*It was survival*,* simply that. Not to feel too deeply … and the uncertainty and the constant question marks… You don’t know how long we’re going to be there… the catastrophes just keep flowing … a feeling that we don’t even have an anchor to give them to lean on. There’s food*,* there’s a place to sleep*,* there are occasional activities*,* but much less parental time.* (Yuli)

Rather than an emotional release, Yuli described a gradual dissociation from her parenting role, echoing a deeper sense of disconnection and inadequacy. Her use of logistical markers: “food,” “place to sleep”, contrasts sharply with the emotional deficits she names: lack of “anchor,” “parental time,” and the pervasive avoidance of feeling. The quote illuminates another contradiction: though the family’s basic needs were met, the emotional core of parenting was eroded by ongoing stress and depletion. These accounts illustrate how, under extreme conditions, parents oscillated between composure and collapse, regulation and overwhelm. Emotional overflows often arrived not as isolated breakdowns but as signals of deeper psychic strain, exposing both the strength and fragility of caregiving during war.

#### Emotional Overflow and Parental Overwhelm: “You Feel Like a Failure as a Mother”

The displacement period introduced a sustained psychological burden that challenged participants’ emotional resilience and caregiving capacities. Three of the mothers interviewed described moments of acute emotional overload and parental inadequacy. These feelings were not isolated breakdowns but cumulative responses to the dissonance between what their children needed and what they felt capable of providing. All participants found themselves navigating their own distress while simultaneously trying to buffer their children from the chaos of war, often without success.

Yafa, for example, described how the strain intensified over time, as the initial novelty of displacement wore off and deeper difficulties surfaced:

*There were outbursts of anger and nerves… At first*,* everything was okay… but when difficulties arose*,* I tried to help and didn’t succeed… My patience*,* I admit… as time passed*,* it became harder… You feel like a failure as a mother.* (Yafa)

Here, Yafa reveals a painful contradiction: her awareness of her children’s emotional deterioration collides with her diminishing capacity to remain present and emotionally regulated. Her repeated use of “I tried… and didn’t succeed” marks a rupture in the caregiving ideal - an internalized standard of maternal efficacy that war conditions made untenable.

Similarly, Rony’s experience in the initial days of the attack highlights how the intensity of her children’s fear clashed with her own emotional paralysis:

*I couldn’t calm him down… he was online all the time and knew more than I even knew… I tried to silence him… I simply couldn’t be there for him… I just told him*,* ‘Be quiet!’* (Rony).

Rony’s attempt to protect her younger daughters from the information overload of her eldest son paradoxically resulted in shutting him down. This moment illustrates a key tension: the competing needs of different children, and the parent’s struggle to manage them while emotionally compromised herself. Her repeated “I couldn’t…” is a refrain of helplessness, underscoring both situational and internal limits.

Yuli, in contrast, emphasized the slow erosion of connection over time, as her son became increasingly emotionally and physically distant: *“A complete loss of control… a feeling of distance… he was a separate entity from us*,* and he felt it too.”* This “loss of control” refers not only to parental authority but to relational intimacy. What began as a survival strategy, trusting an older child to manage independently, mutated into perceived neglect. Later, Yuli articulates this as guilt: *“There was this feeling… like we just pushed him aside*,* because he was older and could manage… but that’s not… it doesn’t make sense.”* Here, the contradiction emerges between reason and emotion: cognitively, she knows the delegation of responsibility was necessary; emotionally, it feels like abandonment. Her repetition of “it doesn’t make sense” suggests an unresolved moral discomfort - what made sense logistically violated her maternal intuition.

#### Fragmented Presence: Caregiving While Absent

In some cases, parents were forced to leave their children for extended periods due to professional emergency roles, compounding their sense of failure and disconnection. Yuli, an ER nurse, recounts how her dual caregiving responsibilities - to both wounded patients and her own children - created impossible trade-offs: *“All day I was in hell at Barzilai (a hospital) … and my husband was fighting on the border… the kids were moving from place to place… under fire.”* Here, the fragmentation is both physical and symbolic: children are shuffled among caregivers while parents are torn between duty and attachment. The image of children “moving… under fire” reflects not just exposure to danger but instability in care - producing what might be called a traumatic dislocation of the caregiving bond itself.

#### Parental Helplessness: “I Was Left Speechless”

Two parents described moments when their children recounted horrific information they had absorbed through peers or digital spaces, before the parents themselves had a chance to process or mediate the content. These moments laid bare the loss of narrative authority and the impossibility of fully protecting children from the psychological violence of war. Miri recalled her young son reporting the death of someone from their community: *“Now*,* I was shocked… I was caught unprepared. I didn’t know what to say… I just said something like*,* ‘No*,* no one died… that’s probably not true…’ And then you’re just left speechless.”* Her denial is not a lie so much as a reflexive attempt to reassert a world in which she can still shield her child from unbearable truths. Yet, this moment also reveals her own inability to regulate the emotional terrain, being “left speechless” becomes emblematic of parental impotence in the face of shared trauma.

Another recurring struggle was how to speak to children about the hostages, a topic that many parents, like Miri, felt unprepared to navigate:

*We decided not to tell them until we knew what was happening… I told only my eldest son*,* but didn’t tell the younger one… there was always the fear of what I was telling them versus what they were hearing from their surroundings… We haven’t managed to have a proper conversation about it.* (Miri)

This reveals another contradiction: silence is meant to protect, but it also leaves a vacuum quickly filled by uncontrolled external narratives. The parent’s role as an emotional interpreter and meaning-maker is undermined by the pervasive, unfiltered presence of war in every sphere of life. The result is a paralysis of language, where even “proper conversations” become elusive.

#### The Double-Edged Sword of the Hosting Community: A Stabilizing Factor in a Disastrous Context

The metaphor of a “double-edged sword” reflects the ambivalent nature of the host community. On the one hand, it provided safety, continuity, and belonging in a disastrous context; on the other, this same communal framework also generated emotional and relational strains that are further elaborated in the following sub-theme. Participants consistently emphasized the critical role of the host community in alleviating the immediate distress of evacuation. For many, relocation to a familiar setting offered a sense of safety, continuity, and belonging amid chaos. Rony, for example, described the host community as an emotionally anchoring environment:

*The hosting village was a place we loved very much. Every time we evacuated*,* we went there. We already had friends there… We knew everyone. It was really like a second home. So it helped a lot. You just want to be somewhere safe*,* to survive*,* you know? You just want everything to be safe … it felt like a recovery center… It was a guesthouse*,* there was grass*,* it was a kibbutz environment*,* and really… we were lucky.* (Rony)

Rony’s account reflects a rare moment of coherence in a disintegrated reality. Her emphasis on familiarity, “we knew everyone” & “It was like a second home”, suggest that social continuity might have served as a protective factor. The metaphor of a “recovery center” further reinforces the idea of the hosting kibbutz as a therapeutic holding environment, where structured activities and pastoral surroundings temporarily soothed the collective nervous system.

#### Challenges of Displacement: Overcrowding, Stress, and Uncertainty

However, the same hosting conditions that offered comfort also generated new stressors. Overcrowding, lack of privacy, and prolonged uncertainty weighed heavily on parents and children alike. Miri, for example, described how the communal living arrangements eroded her sense of home:

*It was absolutely terrible. I couldn’t even say the word ‘home’… No space*,* no living room*,* no ability to have a private family meal*,* you eat in the communal dining hall. Always with everyone*,* always together.* (Miri)

The repetition of “no” signals the negation of family rituals that typically restore order and meaning. Communal living, while necessary, stripped families of basic routines that confer dignity and predictability - core elements of psychological recovery. Yafa echoed this tension, highlighting how physical suffocation mirrored emotional claustrophobia:

*It was suffocating*,* like 360 degrees of suffocation*,* feeling like you had no air. And they had no air either… One of the hardest things was that I couldn’t give him the feeling that there was a solution… You truly live in uncertainty*,* and it is impossible to become accustomed to living in uncertainty.* (Yafa)

Here, the sensory language, “no air”, serves as a visceral metaphor for the limits of parental containment. Yafa not only struggles to manage her own anxiety but also laments her inability to offer her son a sense of stability. The displacement becomes not just a logistical disruption but an existential one.

#### A Refuge That Could not Hold: Ambivalent Safety in the Hosting Community

Though conceived as places of refuge, some hosting communities became sites of secondary trauma. Two participants described how the psychological fragility of fellow evacuees, combined with the recurrence of air raid sirens, intensified their children’s trauma. Yafa recounted one such experience:

*It was terrible and horrific*,* shocking. I can tell you that many years of sirens in the Gaza envelope were not as bad as those two sirens… Because of the number of people*,* and there were a lot of us*,* we were hundreds*,* running together to two shelters*,* all crammed in… with people shouting*,* “Be careful not to lose the children! Watch the children! They’re taking children!” Because people*,* from their own traumatic experiences*,* were not in a good place mentally. My children had never cried during sirens*,* never*,* in their lives. But those two*,* they were shocking. They were the worst. They took a toll*,* one of the truly hardest times*,* with anxiety that stayed with us long after*,* and difficulties*,* and fear of being alone. It was really terrible.* (Yafa)

Yafa’s description complicates the notion of communal support. What should have been protective, “togetherness”, instead amplified the chaos. The crowd dynamics, noise, and shared hypervigilance turned the shelter into a site of collective dysregulation. Her account underscores that proximity to others, especially when unprocessed trauma is widespread, can paradoxically destabilize rather than soothe. The emotional aftermath: lingering anxiety, fear of solitude, points to the long tail of displacement-related trauma, even when physical safety is technically secured.

## Discussion

This study explored the lived experiences of Israeli mothers navigating parenting and disrupted parent-child relationships following the October 7 terrorist attack and subsequent displacement. The findings illuminate the acute psychological burden on parents, shifts in emotional regulation and familial roles within the attachment system and the complex relationship with the hosting community. These themes align with, and in some cases extend, emerging literature on parenting under conditions of armed conflict, cumulative trauma, and forced displacement, by showing how threat and displacement reshape both co-regulation and the wider “ecology of care” surrounding the family (Ungar, 2011).

### Parenting Under Siege: Emotional and Functional Vulnerability

Participants’ narratives revealed profound emotional vulnerability and self-doubt regarding their parenting capacities, particularly when isolated from co-parents, extended family, and familiar social anchors. The mother-participants in the current study described moments of psychological collapse followed by abrupt pivots into action, often for the sake of their children. This emotional pendulum aligns with Mijalevich-Soker and Taubman- Ben-Ari’s (2025) findings that Israeli caregivers during the war vacillated between paralysis and a heightened sense of responsibility, shaped by ongoing uncertainty and disrupted support networks. Parenting in these contexts became an emotional and logistical battleground, where performing strength became both a coping strategy and a perceived parental duty.

Various studies show that heightened regulation alongside internal moral imperatives to remain composed in front of children complicated parent-child dynamic (Mijalevich-Soker & Taubman - Ben-Ari’s, 2025). It seems that caregivers suppress emotional reactions to shield children, thus giving rise to an invisible emotional burden, one that can compromise caregivers’ availability for co-regulation at precisely the moments children most require an “external regulator” (Lieberman & Van Horn, 2005; Lieberman & Horn, 2008).

### Parent-Child Role Shifts and Dependency

Our second theme, “My Child Grew Up Overnight,” captures dramatic shifts in parent-child roles that unfolded under chronic threat and displacement. Across the four interviews, mothers described a paradoxical pairing of *accelerated maturation* (children becoming vigilant, protective, and “hyper-responsible”) alongside *regressive dependency* (clinging, separation distress, sleep disruption, and heightened anxiety). Such dual trajectories, premature independence alongside heightened proximity seeking, have been documented in trauma-exposed families, including under conditions of war-related stress (Feldman & Vengrober, 2011).

From an attachment perspective, these shifts can be understood as adaptations to danger in which children alternate between approach-based bids for co-regulation and more self-reliant strategies when caregiving feels less available (Bowlby, 1969; Taylor & Kaplan, 2023). The children’s apparent “growing up overnight” may also be framed in relation to parent-child role reversal or parentification-like dynamics, in which children assume adult-like vigilance, responsibility, or caregiving functions under conditions of family stress and parental vulnerability. Parentification has been conceptualized as a disturbance in generational boundaries in which children take on emotional and/or functional responsibilities typically held by adults, particularly when parental availability is compromised or the family system is under strain (Boszormenyi-Nagy & Spark, 1973; Earley & Cushway, 2002; Engelhardt, 2012; Macfie et al., 2015). However, in the present study, these dynamics should not be understood as fixed family structures or as formal parentification, but rather as situational, trauma-shaped role shifts emerging under extreme conditions of threat, displacement, and maternal depletion.

From an attachment perspective, such role shifts may affect the child’s sense of the caregiver as a reliably available source of protection. When the child feels required to monitor the parent’s distress or help stabilize the family system, the direction of care can become partially inverted: rather than depending on the caregiver for regulation, the child may attempt to preserve closeness by becoming useful, vigilant, or emotionally attuned to the parent’s needs (Earley & Cushway, 2002; Engelhardt, 2012). Over time, such dynamics may shape the child’s internal working models, reinforcing a sense of self as responsible for others’ emotional stability and a view of relationships as organized around care, duty, and the management of threat (Bowlby, 1969; Earley & Cushway, 2002; Engelhardt, 2012). In the present context, this does not imply a fixed attachment disturbance, but suggests that extreme danger and maternal depletion may temporarily strain the ordinary attachment hierarchy, producing both intensified proximity seeking and premature self-reliance. Importantly, mothers’ narratives suggest that these role shifts were co-shaped by the mothers’ own regulatory capacity under trauma: when mothers felt depleted, emotionally flooded, or fragmented in their caregiving role, children’s strategies appeared to polarize: either stepping into quasi-adult responsibility (to stabilize the household) or intensifying dependency behaviours (to recruit closeness and containment). Thus, the theme does not suggest a simple developmental acceleration, but rather a trauma-shaped oscillation between premature responsibility and intensified dependency.

### The Hosting Community as Both Refuge and Risk

The mothers’ narratives revealed that displacement did not end once physical danger was reduced; rather, it continued to unfold within the relational and communal conditions of the hosting environment. Participants described the hosting kibbutz or temporary settlement as a double-edged context: a place that initially offered safety, continuity, and communal care, yet gradually became associated with overcrowding, tension, loss of privacy, and perceived judgment. The impact of displacement on the hosting environment was emphasized by several researchers (Lozynskyy et al., 2023; Grinshtain & Haruv, 2025; Richmond, 1988). Richmond (1988) discussed how immigrants’ socio-cultural adaptation is shaped by both systemic support and societal conflict in host countries, emphasizing the tensions that arise when integration efforts clash with cultural preservation and societal resistance. Similarly, in the current study, while participants initially experienced the hosting kibbutz as a stabilizing and even therapeutic space, “like a recovery center”, this sense of refuge often eroded over time. The very structures that enabled belonging and continuity also became sources of psychological fragmentation. Cultural expectations, loss of privacy, and cohabitation with other traumatized families intensified distress and undermined parental efficacy. These findings resonate with research on post-disaster social support, which shows that community support may be powerfully protective in the immediate aftermath of trauma, but may also deteriorate or become strained over time when the surrounding community is itself overwhelmed (Kaniasty, 2012).

The Israeli post - October 7 context further clarifies this duality. Recent studies of Israeli evacuees describe displacement not simply as relocation to a safer place, but as a prolonged disruption of autonomy, privacy, family routines, belonging, and community continuity. Assaraf and Dagan (2025), for example, examined evacuees from northern and southern Israel who were displaced to hotels during the Israel–Hamas war, highlighting how even settings intended to provide protection could be experienced as restrictive, liminal, and psychologically distressing.

Ecologically, these accounts can be understood as a shift in the family’s microsystem, where every day “holding environments” (routines, privacy, predictable roles, and social supports) are reconfigured by the host setting and its norms (Beck, 2025; Taylor & Kaplan, 2023). In refugee and displacement research, integration frameworks likewise emphasize that social connections (bonds, bridges, and links), alongside access to services and local attitudes, shape whether host contexts function as protective or stressful environments (Ager & Strang, 2008; Hadar, 2024). From an attachment-informed ecological lens, these pressures seem to reduce caregivers’ capacity to sustain consistent co-regulation and secure-base provision, as parental functioning became continuously exposed to surveillance, comparison, and uncertainty (Mladenović et al., 2021). This is particularly important because hosting communities were not neutral containers of evacuees’ distress; rather, they were themselves embedded within the same national emergency and, in many cases, within an ongoing shared traumatic reality. Literature on shared traumatic reality emphasizes that caregiving, helping, and support are complicated when helpers, professionals, and host environments are exposed to overlapping threats, losses, and uncertainty (Dekel & Baum, 2010; Baum, 2014; Segev et al., 2026).

Grinshtain and Haruv (2025) found evacuees in Israel’s northern periphery describing community displacement as producing both pull and push factors: feelings of detachment, involuntary loss of agency, and deep concern for children’s well-being. The emotional ambivalence reported, safe yet emotionally fragmented, underlines how support must move beyond material needs to address relational disruptions in hosting settings.

### Impacts on Children’s Development and Familial Health

Systematic reviews by Bürgin et al. (2022) and Hazer and Gredebäck (2023) show that forced displacement and parental distress significantly affect young children’s social-emotional and cognitive development, especially when caregivers are overwhelmed or absent. In the current study, these deeply personal accounts of solo parenting during the October 7 attacks and throughout prolonged displacement underscore the compounding psychological burden placed on caregivers, especially mothers, navigating crisis without a co-parent.

The absence of shared decision-making and emotional support intensified feelings of helplessness, guilt, and parental inadequacy. As reflected in Rony and Yafa’s narratives, the dual roles of protector and emotional regulator became both critical and unsustainable under chronic stress.

Alfasi et al. (2025) similarly documented elevated parental stress and its negative impact on family functioning among Israeli parents post-October 7, reinforcing the centrality of caregiver well-being in trauma adaptation. Yet even in the face of exhaustion and fragmentation, the mothers in this study exhibited remarkable efforts to contain their children’s distress, whether through physical proximity, emotional shielding, or moral framing of adversity. This echoes Gillespie et al. (2022), who highlight the critical protective role displaced mothers play in buffering children from complex trauma. Their work emphasizes the urgent need for relational interventions that focus not only on children’s symptoms, but on caregiver capacities and the restoration of parental coherence as the foundation for recovery.

### Toward a Trauma-Informed Model of Displaced Parenting

Taken together, our findings underscore the double burden: mothers navigated trauma both as caregivers and relational regulators. In this context, *caregivers* refer primarily to the practical and protective dimensions of parenting, including securing safety, managing displacement-related demands, and maintaining daily care under conditions of instability. *Relational regulators*, by contrast, refers to mothers’ dyadic emotional role: their efforts to contain children’s fear, sustain emotional availability, and restore co-regulation despite their own traumatic activation. Displacement not only fractured physical stability, but also parental identity and internal regulation capacities, complicating the restoration of a secure base and reliable co-regulation within the parent-child relationship. Recent studies reinforce the importance of reflective functioning and parental efficacy in war-exposed contexts (Enav & Mayer, 2025; Hadar, 2025). This suggests that interventions should strengthen parents’ reflective capacity as a pathway to support both child and caregivers’ resilience. Moreover, Khraisha et al.’s (2024) suggests that redesigned caregiving systems emphasizing the urgency of co-parenting, dignity, negotiation, and gentleness can promote mental health and general family cohesion in the context of displacement.

### Limitations and Future Research

This study is based on in-depth interviews with four mothers who experienced the October 7 attack and subsequent displacement, and its conclusions should therefore be interpreted with caution. The small sample size and the narrow scope of interviewees limit the transferability of the findings; they cannot be assumed to represent the full range of Israeli parents’ experiences across different regions, communities, family structures, or sociocultural backgrounds.

In addition, all participants were mothers recruited through a temporary therapeutic clinic, meaning that their accounts may reflect families who had some degree of access to therapeutic support and were able to reflect on their experiences within a relatively containing context.

A further limitation concerns the cultural and geopolitical specificity of the study. The empirical focus is highly situated within the Israeli context, and more specifically within families from the Israeli periphery who were affected by terror, war, and internal displacement. This specificity is also a strength: it allows for a rich, context-sensitive account of parenting under acute threat and prolonged displacement. However, it also means that the findings should not be generalized uncritically to other settings of war, forced migration, or communal trauma. Parenting under displacement is shaped by local histories, political realities, family structures, cultural expectations, community organization, and available systems of support. These contextual features are central to the meanings participants attributed to solo parenting, children’s accelerated maturation, fragmented caregiving, and the ambivalent role of the hosting community. At the same time, these participants are highly illustrative of a broader phenomenon: mothers parenting under conditions of acute terror, ongoing uncertainty, and prolonged displacement. Their narratives offer rich, conceptually powerful examples of processes that are likely shared by many in similar circumstances, but the study is better understood as providing depth and nuanced insight rather than breadth or statistical representativeness.

Future research should examine parenting under displacement across more diverse cultural, social, and geopolitical contexts. Larger qualitative and mixed-methods studies are needed to explore whether similar patterns emerge among families displaced by different forms of armed conflict, political violence, natural disaster, or forced displacement. Within the Israeli context, future studies should include parents from varied regions, ethnic and religious communities, socioeconomic backgrounds, and family constellations, including fathers, single parents, grandparents, and other caregivers.

Longitudinal and dyadic studies following parents and children over time are also needed, particularly across key transition points such as evacuation, temporary relocation, prolonged hosting, return home, or resettlement elsewhere. Such designs could help clarify how parental representations, child adjustment, attachment-related behaviors, and family routines change over time. They could also examine how hosting environments either support or undermine parental functioning, privacy, autonomy, and family coherence.

## Implications and Recommendations

- Programming

Services working with displaced families (e.g., community centers, host kibbutzim, clinics, schools) should offer brief, structured group interventions for parents that directly target (a) emotion regulation skills (e.g., grounding, co-regulation with children, managing panic in shelters), (b) parental self-efficacy (helping mothers name what they *are* doing well under impossible conditions), and (c) relational coaching (practical strategies for talking with children about fear, absence of the other parent, and uncertainty). These can be delivered as 6–8 session groups or integrated into existing parent meetings and child-focused programs.

- Community-based Support

Hosting environments should be required to build *psychological safety* into their routine operations, not only provide accommodation and food. This may include: orientation meetings that explicitly frame evacuees as valued guests rather than burdens; training for local staff and volunteers on trauma-informed communication (e.g., avoiding judgmental comments about parenting); quiet, private spaces where parents can decompress without feeling exposed; and regular community forums where tensions can be voiced and mediated rather than left to simmer.

- Policy

Resettlement and internal displacement policies should prioritize (a) maintaining family unity where possible (avoiding unnecessary separations of parents and children across different sites), (b) funding on-site, low-barrier childcare and early childhood development programming within temporary housing, and (c) embedding mental-health - informed parental support (e.g., psychoeducational groups, parent counselling) into national emergency response packages (Bernhardt et al., 2024). Policy guidelines can specify minimum standards for psychosocial support in hotels, kibbutzim, and temporary centers, and earmark budgets for employing trained psychosocial staff rather than relying solely on volunteers.

## Conclusion

This study sheds light on the lived experiences of Israeli mothers navigating parenting under conditions of extreme terror and prolonged displacement. Across the interviews, parenting emerged as both a practical and deeply relational task, shaped by the simultaneous demands of protection, emotional regulation, role negotiation, and survival. The findings show how traumatic crisis and displacement can fracture not only physical homes and daily routines, but also parental identity, emotional coherence, and the parent–child relational field. Mothers were required to maintain safety and continuity for their children while managing their own fear, grief, exhaustion, and disorientation. In this context, caregiving became an ongoing act of emotional containment, often requiring mothers to project stability at moments when their own internal sense of stability had been profoundly disrupted.

The study highlights the need for trauma-informed responses that recognize displaced parents not only as caregivers responsible for children’s recovery, but also as survivors whose own emotional and relational capacities require support. Clinical and community-based interventions should therefore attend to the family system as a whole, strengthening relational safety, parental reflective capacity, emotional regulation, and opportunities for parental agency and dignity. Supporting children in the aftermath of war and displacement cannot be separated from supporting the parents who are asked to hold, interpret, and regulate the traumatic reality for them. Recovery, in this sense, involves more than restoring routines or providing temporary refuge; it requires attention to the fractured emotional and relational structures through which families attempt to regain continuity, connection, and a sense of safety.

## Data Availability

In order to protect participants’ confidentiality and in line with the conditions set by the institutional ethics committee, the data collected for this study are kept securely by the authors and are not available for sharing.

## Footnotes

1 Emotional regulation refers to the processes through which individuals monitor, understand, and modulate their emotional states in order to respond adaptively to internal and external demands. In early childhood, this capacity develops largely through co-regulation with caregivers, who help the child organize distress, restore calm, and gradually internalize strategies for managing affect (Paley & Hajal, 2022).

2 Attachment refers to the child’s enduring emotional bond with a caregiver who is experienced as a source of protection, comfort, and regulation in times of distress. Through repeated interactions, the child develops expectations about whether others are available and responsive, shaping later patterns of safety, exploration, affect regulation, and relationships (Bowlby, 1969).

3 In this article, solo parenting refers to periods in which a parent carries primary day-to-day responsibility for the children without the physical presence of a co-parent. The term does not necessarily indicate formal single-parent status. This experience became particularly relevant in Israel following the October 7 attack due to displacement and other war-related separations, including prolonged reserve military service. By January 2024, approximately 300,000 reservists had been called up, around 120,000 of whom were parents; approximately 12% of those mobilized were parents of children under the age of nine (Shay et al., 2024; Zerach, 2024).

## Declarations

### Declaration of AI-Assisted Editing

The author acknowledges the use of ChatGPT for language editing and stylistic refinement during the preparation of this manuscript. The tool was used solely to improve clarity, grammar, and readability. All intellectual content, analysis, interpretation, and final decisions regarding the manuscript remain the responsibility of the authors.
